# VGLL4 targets a TCF4–TEAD4 complex to coregulate Wnt and Hippo signalling in colorectal cancer

**DOI:** 10.1038/ncomms14058

**Published:** 2017-01-04

**Authors:** Shi Jiao, Chuanchuan Li, Qian Hao, Haofei Miao, Lei Zhang, Lin Li, Zhaocai Zhou

**Affiliations:** 1 State Key Laboratory of Cell Biology, CAS Center for Excellence in Molecular Cell Science, Institute of Biochemistry and Cell Biology, Shanghai Institutes for Biological Sciences, Chinese Academy of Sciences, Shanghai 200031, China; 2 The School of Life Science and Technology, ShanghaiTech University, Shanghai 200031, China; 3 State Key Laboratory of Molecular Biology, CAS Center for Excellence in Molecular Cell Science, Institute of Biochemistry and Cell Biology, Shanghai Institutes for Biological Sciences, Chinese Academy of Sciences, Shanghai 200031, China

## Abstract

Concerted co-regulation of multiple signalling pathways is crucial for tissue homoeostasis and tumorigenesis. Here we report that VGLL4, a previously identified YAP antagonist, also functions as a regulator of Wnt/β-catenin signalling. The expression of VGLL4 is significantly downregulated in clinical colorectal carcinoma (CRC) specimens, positively associated with patient survival rate, and inversely correlated with the expression of Wnt target genes in CRCs. Knockdown of VGLL4 enhances proliferation and tumour formation of CRC cells. A designed peptide mimicking the function of VGLL4 effectively inhibits CRC progression in a *de novo* mouse model. Mechanistically, TEAD4 associates with TCF4 to form a complex and cobind target genes. VGLL4 targets this TEAD4–TCF4 complex to interfere the functional interplay between TEAD4 and TCF4, suppressing the transactivation of TCF4. Collectively, our study indicates that Wnt/β-catenin and Hippo-YAP signalling are directly linked at transcription factor-level, and VGLL4 can target a TEAD4–TCF4 complex to co-regulate both pathways.

## Abstract (web summary)

The Wnt/β-catenin and YAP signaling pathways have fundamental roles in cancer. Here, the authors show that VGLL4, a known YAP antagonist, also negatively regulates Wnt/β-catenin signaling by targeting TEAD-DNA-TCF4 complex, thereby inhibiting colorectal cancer growth.

The evolutionarily conserved Wnt/β-catenin and Hippo-YAP signalling pathways play fundamental roles in individual development and tissue homoeostasis^1^,^2^,^3^,^4^,^5^,^6^,^7^. A shared core feature of the Wnt/β-catenin and Hippo-YAP signalling pathways is the phosphorylation-dependent control of a key transcriptional co-activators, namely the regulation of the level and nuclear localization of β-catenin and YAP/TAZ, respectively^8^,^9^,^10^. Specifically, β-catenin is retained in the cytoplasm and undergoes degradation in the off state of Wnt signalling; while the retention and degradation of YAP/TAZ occur in the on state of Hippo signalling. When the Wnt signalling is switched on, β-catenin translocates into the nucleus, where it interacts with the transcription factors TCF4/LEF1 to regulate the expression of the target genes. Similarly, when Hippo signalling is switched off, YAP/TAZ accumulates in the nucleus where it interacts with the TEA domain (TEAD) family transcription factors (TEAD1-4 in mammals) to control target gene expression. Thus, the activities of the oncogenic effectors β-catenin and YAP/TAZ need to be precisely regulated to ensure balanced cell growth and tissue homoeostasis.

Dysregulation of Wnt/β-catenin or Hippo-YAP signalling pathways has multiple pathological consequences. For example, >90% of colorectal cancer (CRC) patients show aberrant activation of the Wnt/β-catenin signalling pathway, resulting in sustained accumulation of β-catenin in the nucleus, and suggesting that transactivation of β-catenin-TCF4 target genes represents a primary initial event in CRC (ref. 11). Other mutations of the Wnt/β-catenin pathway that lead to its constitutive activation were found in gastric cancer, bone cancer, hepatocellular carcinoma, medulloblastoma, breast cancer and ovarian cancer^7^,^12^. In contrast, mutations in components of the Hippo-YAP signalling pathway are rare. Nevertheless, elevated activity of YAP/TAZ has been extensively correlated with various cancers including lung^13^,^14^, colorectal^15^,^16^, breast^17^, ovarian^18^, liver^19^,^20^ and prostate cancers^21^. Despite the clear association of Wnt/β-catenin and Hippo-YAP signalling with various cancers, targeted therapies aiming at these pathways remain limited^22^.

There is a growing amount of evidence for multi-point crosstalk between the Wnt/β-catenin and Hippo-YAP signalling pathways. Most studies to date indicate that YAP/TAZ can act as direct mediators between these pathways. For example, the Hippo-YAP pathway has been reported to be involved in the regulation of Wnt/β-catenin signalling through the interaction of YAP/TAZ with β-catenin and/or DVL (refs 23, 24). In particular, the absence of the Hippo-YAP pathway component *SAV* results in robust transcriptional upregulation of Wnt/β-catenin target genes^25^. It was suggested that in this case YAP-TEAD and β-catenin-TCF4 act cooperatively to promote the expression of *sox2* and *snail2*. Recently, Piccolo and colleagues proposed that YAP/TAZ could be a constitutive component of the β-catenin destruction complex^26^. They proposed that any retention of YAP/TAZ in the cytoplasm would promote destruction complex activity and inhibit Wnt/β-catenin signalling; in contrast, translocation of YAP/TAZ into the nucleus and away from the destruction complex would activate Wnt/β-catenin signalling. According to these findings, phosphorylated β-catenin is required for YAP/TAZ to associate with the β-TrCP E3 ligase complex; while YAP/TAZ can also recruit β-TrCP to the β-catenin destruction complex^26^. Stimulation by Wnt could release both β-catenin and TAZ from the destruction complex, leading to gene transcription mediated by TCF4 and TEAD.

Cooperation between transcription factors is a common theme, and DNA-dependent formation of transcription factor pairs can alter their binding specificity^27^. However, the potential cooperation between TCF4 and TEAD4, transcription factors of the Wnt/β-catenin and Hippo-YAP signalling pathways, remain poorly understood. It is also unclear whether other binding partners of TEAD4, such as VGLL proteins play a role in the co-regulation of Wnt/β-catenin and Hippo-YAP signalling. Previously, we and others identified VGLL4 as a transcriptional suppressor that inhibits YAP-induced overgrowth and tumorigenesis through direct competition with YAP for binding TEAD4 (refs 28, 29, 30, 31). Based on a mechanistic dissection, we further developed a peptide that mimics VGLL4 function to treat gastric tumours.

In this work, we report the clinical relevance of VGLL4 in CRC and its regulatory effect in the Wnt/β-catenin signalling pathway. The expression of VGLL4 is significantly downregulated in CRC patients, and inversely correlated with the expression of Wnt/β-catenin target genes. Loss of VGLL4 is associated with decreased survival rates of CRC patients, highlighting a critical role for VGLL4 in tumorigenesis. Surprisingly, the Hippo pathway transcription factor TEAD4 directly associates with the Wnt pathway transcription factor TCF4 via their DNA-binding domains, forming a complex on target genes. VGLL4 binds to this TEAD4-TCF4 complex to inhibit transactivation of both TCF4 and TEAD4. These findings not only identify VGLL4 as a coregulator of both Wnt/β-catenin and Hippo-YAP signalling, but also reveal a functional interplay between the transcription factors of these two pathways.

## Results

### VGLL4 as a diagnostic/prognostic marker for CRC

**Figure 1:**
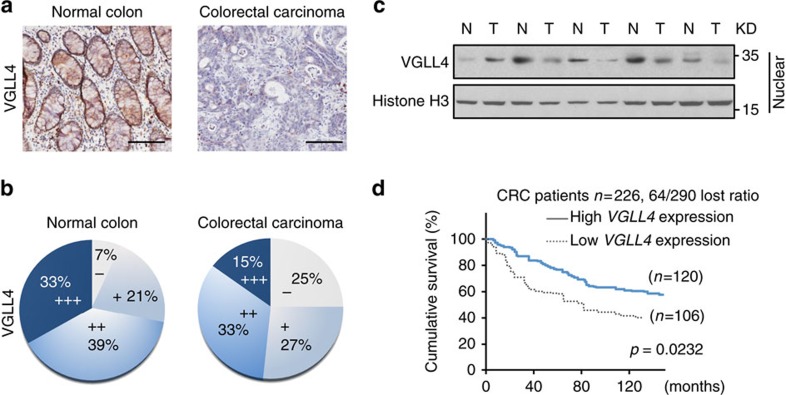
VGLL4 levels are decreased in the CRC samples. (**a**) Representative images of VGLL4 staining on tissue microarray. Scale bar denotes 100 μm. (**b**) VGLL4 staining levels of normal and cancerous colon tissue indicating negative (−), weak (+), moderate (++), strong (+++) VGLL4 expression levels. (**c**) Levels of VGLL4 protein in five human colorectal carcinomas and their paired adjacent normal tissues were analysed. (**d**) Kaplan–Meier survival analysis of patients with high (*n*=226) or low (*n*=106) VGLL4 mRNA levels from GSE14333 dataset by using the logrank test. Survival curves were calculated according to the Kaplan–Meier method. *P*<0.05 was considered to indicate a significant difference. See also Supplementary Fig. 1.

To investigate a potential role of VGLL4 in CRC tumorigenesis, we performed immunohistochemistry studies in a cohort of 40 human patients containing normal colons and 60 patients with CRCs to examine the expression profiles of VGLL4. Interestingly, we found that 72% (29 out of 40) of patients with normal colons exhibited high levels of nuclear VGLL4 (++ and +++) in their colons, whereas only 48% (29 out of 60) of patients with CRC had high nuclear expression of VGLL4 in the cancerous samples from their colons (Fig. 1a,b). The pattern of VGLL4 expression was statistically different between normal colon tissue and CRCs (*P*<0.01). Moreover, VGLL4 staining was significantly associated with tumour size, lymph node metastasis and TNM stage (*P*<0.05), but not significantly correlated to age, gender and tumour metastasis (Table 1). Consistently, immunoblot analysis undertaken for five pairs of CRC samples further demonstrated much lower levels of VGLL4 protein in CRC tissues than in paired normal tissues (Fig. 1c). Taken together, these results indicated that VGLL4 is downregulated in CRC tissues.

To explore the role of VGLL4 as a tumour suppressor with more rigorous clinical data and with more CRC samples, the colon cancer patient dataset GSE14333 available in the Gene Expression Omnibus (GEO) Database (http://www.ncbi.nlm.nih.gov/gds) was employed^32^. We found that the messenger RNA (mRNA) levels of *VGLL4* were negatively correlated with tumour stage (Supplementary Fig. 1). Moreover, low *VGLL4* mRNA levels were associated with shorter survival (Fig. 1d). Altogether, these analyses suggest that VGLL4 could be used as a diagnostic/prognostic marker for CRC.

### Inverse correlation of VGLL4 with Wnt and Hippo target genes

Since VGLL4 was previously identified as a YAP antagonist, we therefore examined the expressions of YAP and its target genes in CRC. As expected, YAP was significantly upregulated, which was accompanied by increased expression of its target genes *CTGF*, *CYR61* and *CDX2* (Supplementary Fig. 2a–d). The expressions of YAP and *CTGF* in high tumour stages were significantly higher than those in low tumour stages (Supplementary Fig. 2e). Moreover, Spearson analysis revealed that the expression of VGLL4 was negatively correlated with those of YAP and *CTGF* (Supplementary Fig. 2f). Similar observations were obtained by immunoblotting analysis (Supplementary Fig. 2g).

**Figure 2:**
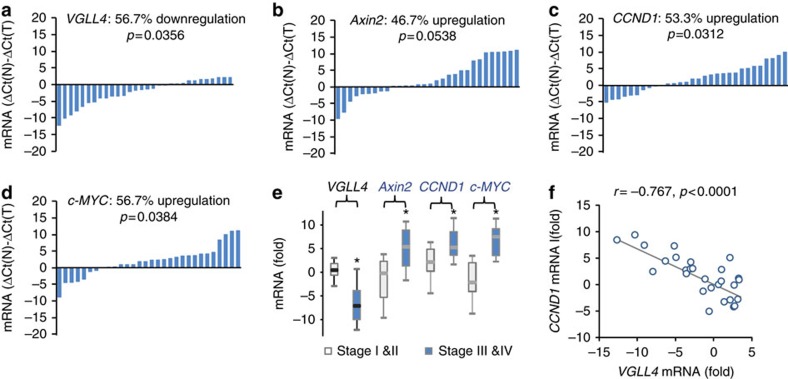
Downregulation of VGLL4 is associated with upregulation of Wnt target genes. (**a**) *VGLL4* mRNA levels in CRC. Levels of VGLL4 mRNA in 30 paired human CRC and normal tissues were examined by real-time PCR, using *GAPDH* as an internal control. The value of each bar shows the difference between the *VGLL4* mRNA level in normal tissue and that in the tumour, and is represented by the equation Δ*Ct*(*N*)−Δ*Ct*(*T*): a value>1 indicates that the *VGLL4* mRNA level is increased in the tumours, and value<−1 indicates that the *VGLL4* mRNA level is decreased in the tumours. Experiments were repeated two times. Paired *t* test was used to compare between paired tumour and normal tissues. (**b**–**d**) mRNA levels of Wnt target genes in CRC tissue. Relative mRNA levels of *Axin2* (**b**), *CCND1* (**c**) and *c-MYC* (**d**) were analysed. Experiments were repeated two times. Paired *t* tests were used. (**e**) Box plots of *VGLL4* and Wnt target gene levels at different tumour stages. Data are expressed as median and interquartile range. Experiments were repeated two times. Unpaired *t* tests were used. *P<0.05, significant relative to control. (**f**) Scatter plot between *VGLL4* and *CCND1* mRNA levels in CRC samples. *VGLL4* expression was compared with *CCND1* by applying Spearman's correlation. Experiments were repeated two times (Supplementary Fig. 2).

Given the close association between Wnt/β-catenin signalling and CRC tumorigenesis, we next examined a potential correlation between VGLL4 and Wnt/β-catenin target genes by comparing their transcription levels in paired CRC tissues derived from the same patient (*n*=30). A univariate analysis (paired *t* test) indicated that the mRNA levels of VGLL4 were decreased in 17 out of 30 (57%) CRC samples relative to normal tissue (*P*=0.0356) (Fig. 2a), confirming our above observations at the protein level. Consistent with the previous reports in CRC samples^33^,^34^, we also found that transcription of downstream target genes of Wnt signalling, including *CCND1* and *c-MYC*, increased markedly in tumour samples, whereas *Axin2* was somewhat, but not significantly, upregulated in the CRC samples (Fig. 2b–d).

Furthermore, a hierarchical cluster analysis revealed that the mRNA levels of *CCND1*, *Axin2* and *c-MYC* significantly increased as the cancer progressed, whereas the mRNA levels of *VGLL4* at the late stage of CRC (stage III and IV) were significantly less than the mRNA levels at the early stage (stage III and IV) of the disease (Fig. 2e). Moreover, downregulation of *VGLL4* mRNA levels was correlated with upregulation of *CCND1* transcription (Fig. 2f). Taken together, these observations indicate an inverse correlation between the transcription of VGLL4 with that of Wnt and Hippo target genes.

### VGLL4 negatively regulates TCF4 and TEAD4 transactivation

**Figure 3:**
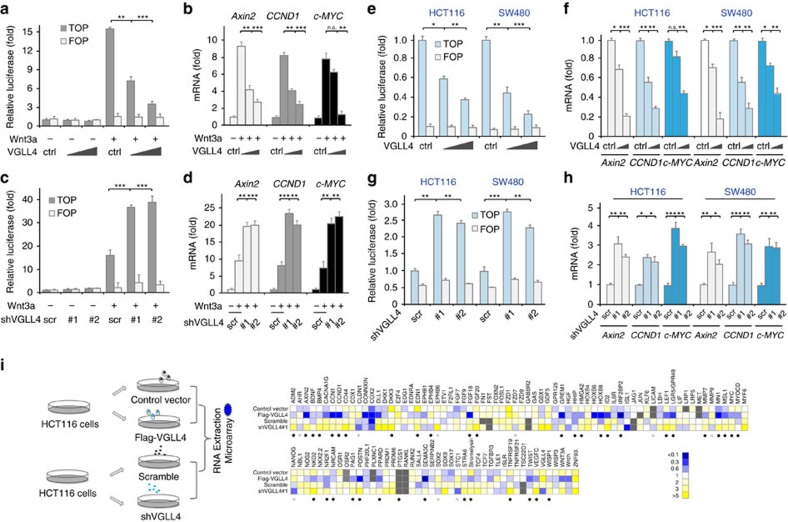
VGLL4 inhibits Wnt/β-catenin signalling. (**a**) VGLL4 inhibited in a dose-dependent manner TOP-FLASH activity in HEK293T cells treated with Wnt3a. (**b**) VGLL4 inhibited transcription of Wnt target genes in HEK293T cells treated with Wnt3a. (**c**,**d**) Knockdown of VGLL4 promotes TOP-FLASH activity (**c**) and transcription of Wnt target genes (**d**) in HEK293T cells. (**e**) VGLL4 inhibits TOP-FLASH activity in HCT116 and SW480 cells. (**f**) VGLL4 inhibits transcription of Wnt target genes in HCT116 and SW480 cells. (**g**,**h**) Knockdown of VGLL4 promotes TOP-FLASH activity (**g**) and transcription of Wnt target genes (**h**) in HCT116 and SW480 cells. (**i**) Microarray analysis of Wnt target genes' transcription in HCT116 cells after transfection with VGLL4 or shVGLL4. Genes negatively regulated by VGLL4 are marked by black dots; while gene positively regulated by VGLL4 are marked by grey dots. *Note:* Data in **a**–**h** represent the mean±S.D. from one experiment. Experiments we repeated three times. Unpaired *t* tests were used to compare the differece between groups. *P<0.05, **P<0.01, ***P<0.001, significant relative to control. n.s., no statistics significance; ctrl, control vector (same below); scr, scramble shRNA (same below) (Supplementary Figs 3–5).

To investigate the role of VGLL4 in Wnt/β-catenin signalling, we performed a β-catenin/TCF4 luciferase reporter (TOP-FLASH) assay in 293T cells. As shown in Fig. 3a, ectopic expression of VGLL4 inhibited Wnt3a-induced TOP-FLASH luciferase reporter activity in a dose-dependent manner. In contrast, overexpression of VGLL4 had no significant effect on FOP-FLASH luciferase activity in which the β-catenin/TCF4 responsive elements were mutated and inactive (Fig. 3a). Consistent with these observations, transfection of VGLL4 in 293T cells reduced in a dose-dependent manner Wnt3a-induced expression of *Axin2*, *CCND1* and *c-MYC*, which are target genes of Wnt/β-catenin pathway implicated in various tumours (Fig. 3b). These results suggest a negative role for VGLL4 in Wnt/β-catenin signalling.

To verify the potential inhibitory role of VGLL4 in Wnt/β-catenin signalling, we next used two independent small hairpin (sh) RNA vectors that efficiently knocked down human VGLL4 expression (Supplementary Fig. 3a). In contrast with the effect of VGLL4 overexpression, depletion of endogenous VGLL4 in 293T cells significantly increased Wnt3a-induced activity of the TOP-FLASH luciferase reporter, as well as mRNA levels of Wnt target genes *Axin2*, *CCND1* and *c-MYC*, again suggesting an inhibitory role of VGLL4 in the Wnt/β-catenin pathway (Fig. 3c,d).

As Wnt3a-induced nuclear accumulation of β-catenin is a hallmark for the activation of the Wnt/β-catenin pathway, we next examined whether VGLL4 could affect the cytosolic/nuclear localization of β-catenin upon Wnt3a stimulation in HEK293T cells. Western blotting analysis of nuclear and cytosolic β-catenin revealed that VGLL4 did not significantly affect Wnt3a-induced translocation of β-catenin to the nucleus (Supplementary Fig. 3b). Consistent with this observation, staining of β-catenin also showed a marginal effect of VGLL4 on Wnt3a-stimulated enrichment of nuclear β-catenin (Supplementary Fig. 3c). These data suggest that VGLL4 does not mainly target the nuclear translocation of β-catenin during its inhibition of Wnt/β-catenin signalling.

Subsequently, we examined the effect of VGLL4 on Wnt/β-catenin signalling in human colorectal cancer cell lines HCT116 and SW480. These cells harbour mutations of *CTNNB1* or *APC*, therefore leading to constitutive activation of the Wnt/β-catenin pathway. Similar to the observations in Wnt3a-stimulated 293T cells, the TOP-FLASH luciferase reporter activity and transcription of Wnt target genes (*Axin2*, *CCND1* and *c-MYC*) were decreased by overexpression of VGLL4, but increased by knockdown of VGLL4 (Fig. 3e–h). Meanwhile, we observed that VGLL4 negatively regulated YAP-induced TEAD4 transactivation and its target gene's transcription in HCT116 and SW480 cells (Supplementary Fig. 4). Taken together, these results clearly indicate that VGLL4 functions as a negative regulator of both TCF4 and TEAD4 transactivation in CRC.

Furthermore, to corroborate the above observations and assess the effect of VGLL4 on Wnt target genes at a genome-wide level, we performed microarray analysis with VGLL4 overexpression or knockdown in HCT116 cells (Fig. 3i; Supplementary Fig. 5a). Among 19992 genes detected in HCT116 cells, 305 genes were negatively regulated by VGLL4, that is, 1.5% of all expressed genes were negatively regulated by VGLL4 (Supplementary Fig. 5b). Meanwhile, among the 114 Wnt target genes (http://web.stanford.edu/group/nusselab/cgi-bin/wnt/target_genes), 39 genes were negatively regulated by VGLL4, that is, 34.2% of Wnt target genes were negatively regulated by VGLL4 (Fig. 3i), suggesting a specific regulatory effect of VGLL4 on Wnt pathway. In addition, gene-ontology analysis revealed that 6 key genes (DKK3, CCND1, WISP1, NKD2, LEF1, AXIN2) involved in Wnt signalling, 9 genes in cell proliferation and 7 genes in apoptosis were regulated by VGLL4, and that Wnt signalling pathway was enriched the most (Supplementary Fig. 5c,d). Again, these results confirm that VGLL4 function as a key modulator in Wnt signalling.

### Inhibition of Wnt signalling by VGLL4 is dependent on TEAD4

**Figure 4:**
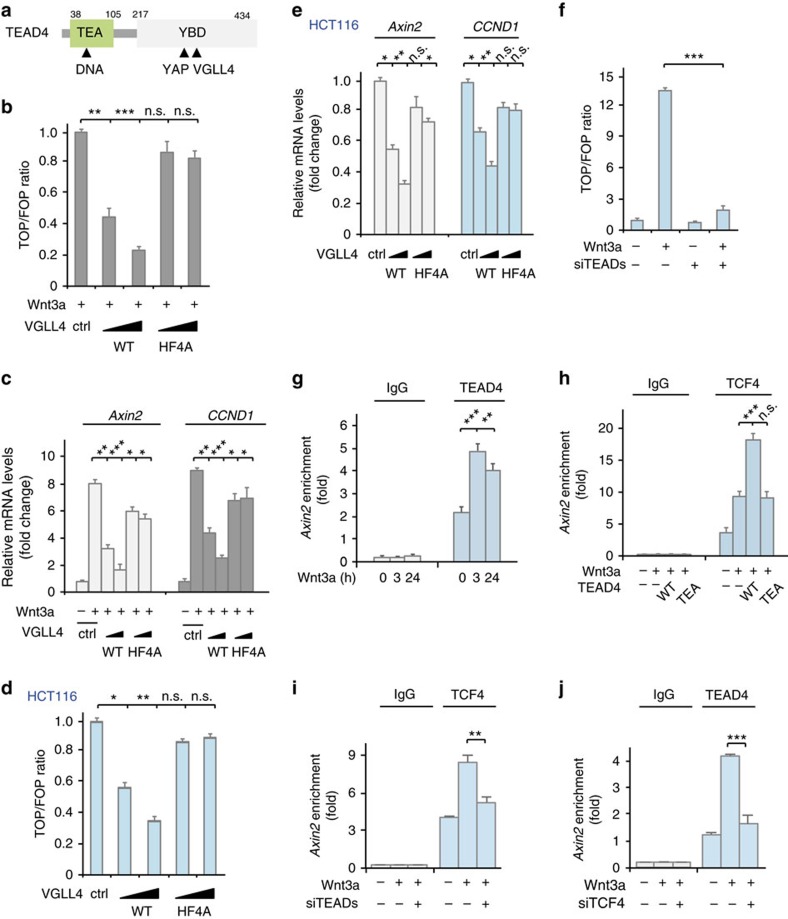
VGLL4 inhibition of Wnt signalling depends on a TEAD4-TCF4 interplay. (**a**) Schematic illustration of the domain organization for human TEAD4. Amino acid numbering is according to human TEAD4. (**b**,**c**) A VGLL4 mutant (HF4A) defective in binding TEAD failed to inhibit TOP-FLASH activity (**b**) and transcription of Wnt target genes (**c**) in HEK293T cells treated with Wnt3a. (**d**,**e**) The VGLL4 mutant (HF4A) did not inhibit TOP-FLASH activity (**d**) and transcription of Wnt target genes (**e**) in HCT116 cells. (**f**) Knockdown of TEADs (TEAD1, 2, 3, 4) inhibited TOP-FLASH reporter activity in Wnt3a-treated HEK293T cells. (**g**,**h**) Chromatin was immunoprecipitated with the TEAD4 antibody (**g**) or TCF4 antibody (**h**) followed by qPCR using primer pairs spanning the human Axin2 locus. Results are presented as percentage immunoprecipitated over input and are representative of three independent experiments. (**i**,**j**) ChIP-qPCR showing TCF4/TEAD4 binding to the Axin2 promoter in 293T cells transfected with control siRNA, TCF4 siRNA (siTCF4s) or TEADs siRNAs (siTEADs) with the TCF4 antibody (**i**) or TEAD4 antibody (**j**). *Note:* Data in **b**–**j** represent the mean±s.d. from one experiment. Experiments we repeated three times. Unpaired *t* tests were used to compare the difference between groups. *P<0.05, **P<0.01, ***P<0.001, significant relative to control. n.s., no statistics significance. HF4A, a VGLL4 mutant in which amino acids H212, F213, H240 and F241 were substituted with alanines. GFP-TEA, GFP-tagged TEAD4 TEA domain (Supplementary Fig. 6).

Members of the TEAD family transcription factors TEAD1-4 (TEADs) contain an N-terminal TEA domain responsible for DNA binding, and a C-terminal YAP-binding domain (YBD) responsible for binding YAP/TAZ and VGLL proteins (Fig. 4a). Since VGLL4 can directly bind to the YBD domain of TEADs, we then asked whether a VGLL4–TEADs interaction is required for its suppressive role in the Wnt/β-catenin signalling. To test this possibility, we took advantage of our previous crystallographic analysis of the VGLL4–TEAD4 complex to design a VGLL4 mutant (H212A/F213A/H240A/F241A, termed as HF4A) unable to bind TEAD4 (ref. 31). As shown in Fig. 4b,c, wildtype VGLL4 significantly inhibited Wnt3a-stimulated TOP-flash reporter activity and the transcription of Wnt target genes *Axin2* and *CCND1* in HEK293T cells; while the VGLL4 mutant almost completely lost its suppressive effect on Wnt/β-catenin signalling. Similarly, transfection of wildtype VGLL4, but not its mutant defective in binding TEAD4 inhibited in a dose dependent manner the TOP-flash reporter activity and transcription of target genes in HCT116 cells (Fig. 4d,e). Altogether, these observations suggest that the interaction of VGLL4 with TEAD could play a role in VGLL4-mediated inhibition of Wnt/β-catenin signalling.

If the interaction of VGLL4 with TEADs is essential for VGLL4-mediated negative regulation of TCF4 transactivation, one may expect that TEADs could play an important role in Wnt/β-catenin signalling. To verify the functional relevance of TEADs in Wnt/β-catenin signalling, we performed TOP-FLASH luciferase reporter assay in cells depleted of TEAD1-4 (Supplementary Fig. 6a). Notably, knockdown of TEADs almost completely abolished Wnt3a-induced transactivation of the TOP-FLASH luciferase reporter (Fig. 4f). This observation indicates that TEADs play a key role in Wnt/β-catenin signalling and that VGLL4 may inhibit TCF4 transactivation through direct binding to TEADs.

Since the TOP-FLASH luciferase reporter plasmid does not contain classical TEAD4 consensus binding sites, and that VGLL4 can inhibit TOP-FLASH reporter activity in a TEAD4 dependent manner, we reasoned that TEAD4 may regulate the transactivation of TCF4 in a noncanonical manner. Given that Hippo-YAP signalling was previously proposed to cooperate with Wnt/β-catenin signalling during heart development^25^, we hypothesized that TEAD4 may regulate Wnt signalling via direct cooperation with TCF4 on the promoter region of target genes. To test this possibility, we first detected the binding of TEAD4 to the promoter of Wnt target gene Axin2 by ChIP assay in Wnt3a-treated 293T cells using an antibody against TEAD4. Our result showed that TEAD4 but not the control protein IgG was bound to the promoter region of Axin2, and Wnt3a stimulation increased such interaction (Fig. 4g). Next, we examined whether TEAD4 could affect the association of TCF4 with Axin2 promoter. As expected, TCF4 but not the control protein IgG could bind to the Axin2 promoter (Fig. 4h). Importantly, overexpression of wildtype TEAD4 but not a TEA domain truncation mutant significantly enhanced the association of TCF4 with Axin2 promoter (Fig. 4h). Moreover, knockdown of TEADs obviously decreased TCF4 occupancy on the Axin2 promoter region (Fig. 4i). Meanwhile, depletion of TCF4 also significantly impaired Wnt3a-induced TEAD4 binding to the promoter region of *Axin2* (Fig. 4j), indicative of a functional interplay between TEAD4 and TCF4. These observations were further confirmed by ChIP assay for a set of (>10) Wnt/β-catenin target genes including LEF1, MYC, DKK1, CCND1 and FGF20 (Supplementary Fig. 6b).

Taken together, these results suggest that TEAD4 may facilitate TCF4 transactivation by co-binding to target genes, and that TCF4 and TEAD4 are inter-dependent for binding to the promoter regions. VGLL4 could target the functional interplay between TEAD4 and TCF4 to suppress the transactivation of TCF4.

### VGLL4 targets a TEAD4-TCF4 complex to inhibit Wnt signalling

**Figure 5:**
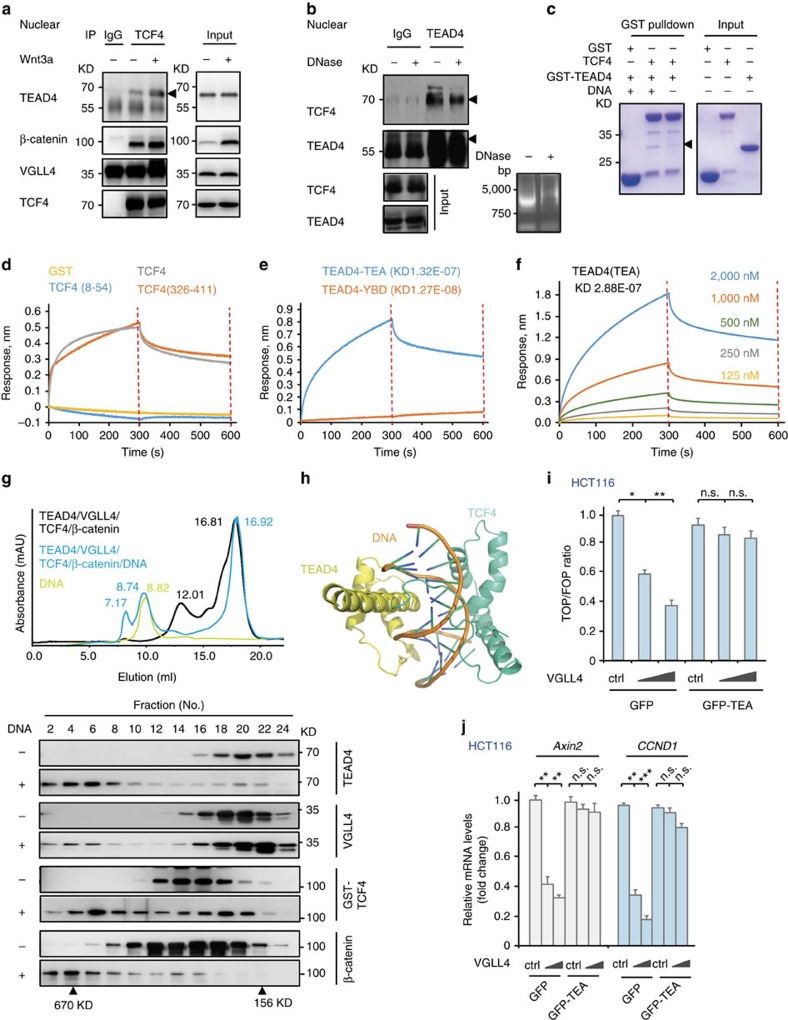
TEAD4 and TCF4 forms a complex for to cobind target genes. (**a**) Coimmunoprecipitation to analyse the association of TEAD4 and TCF4. HEK293T cells were treated with or without Wnt3a for 4 h. Then the cell lysates were incubated and immunoprecipitated with anti-TCF4 antibody or control IgG. (**b**) Co-IP assay to determine the interaction of TEAD4 and TCF4 with or without DNAse. Digestion of DNA was monitored by agarose gel. (**c**) Pulldown assay to detect the TEAD4-TCF4 interaction using purified recombinant proteins with or without a DNA fragment of SOX2 promoter. (**d**–**f**) Biolayer Interferometry analysis of interactions between TCF4 and TEAD4 using purified recombinant proteins. (**d**) Full length TEAD4 (coated on chip) can bind to GST-tagged full length TCF4 or GST-tagged TCF4 (amino acids 326–411), but not GST-tagged TCF (amino acids 8–54) or GST. (**e**) Full length TCF4 (coated on chip) can bind to the TEA domain but not the YBD of TEAD4. (**f**) TCF4 (amino acids 326–411) (coated on chip) can bind to the TEA domain of TEAD4 in a dose-dependent manner. (**g**) Gel filtration analysis of interactions among VGLL4, TEAD4, TCF4 and β-catenin with or without the SOX2 DNA. (**h**) Structure modelling of the interaction of TCF4 in complex with TEAD4 and DNA. (**i**,**j**) Overexpression of the TEAD4 TEA domain abrogated VGLL4-mediated inhibition of TOP-FLASH reporter activity (**i**) and transcription of Wnt target genes (**j**) in HCT116 cells. Data in **i** and **j** represent the mean±s.d. from one experiment. Experiments we repeated three times. Unpaired *t* tests were used to compare the difference between groups. *P<0.05, **P<0.01, ***P<0.001, significant relative to control. n.s., no statistics significance.

To further dissect the mechanism of VGLL4-TEAD4-mediated inhibition of Wnt/β-catenin signalling, we examined a potential physical association of VGLL4-TEAD4 with TCF4. Surprisingly, co-immunoprecipitation assay in 293T cells showed that endogenous TEAD4 and VGLL4 associated with TCF4, as did the positive control β-catenin (Fig. 5a). Wnt3a stimulation significantly enhanced the interaction of TEAD4 with TCF4 (Fig. 5a), which is consistent with the above observation that TEAD4 and TCF4 co-bind to the promoter region of Wnt/β-catenin target genes upon Wnt3a treatment. Meanwhile, TCF4 can also be immunoprecipitated by TEAD4 (Fig. 5b). Moreover, adding DNAase in the IP assay significantly decreased the association of TEAD4 with TCF4 (Fig. 5b), suggesting that such interaction could be enhanced by DNA. Further *in vitro* pulldown assay using purified recombinant proteins of TEAD4 and TCF4 revealed a direct interaction between TEAD4 and TCF4 (Fig. 5c, lane 3). Addition of the promoter region of *SOX2*, a target gene that could be coregulated by TEAD4 and TCF4 (ref. 25) further increased the interaction between TEAD4 and TCF4 (Fig. 5c). Subsequently, these observations were further confirmed by biolayer interferometry analysis using purified recombinant proteins of TEAD4 and TCF4. As shown in Fig. 5d, TEAD4 can bind to full-length TCF4 or its DNA-binding domain (amino acids 326–411) but not the β-catenin-binding domain (amino acids 8–54) or the control protein GST. On the other hand, the TEA domain of TEAD4 can bind to TCF4; while the YBD domain of TEAD4 could not bind to TCF4 at the same condition, suggesting a specific interaction between the DNA binding domains of TEAD4 and TCF4 (Fig. 5e). Indeed, the purified recombinant protein of the TEAD4 TEA domain can bind in a dose-dependent manner to the purified recombinant protein of the DNA binding domain of TCF4 with a sub milli-molar Kd (Fig. 5f). Consistent with this result, gel filtration chromatography showed that VGLL4-TEAD4 and TCF4-β-catenin were partially co-eluted; while in the presence of DNA, all four proteins were largely co-eluted in a peak with much higher molecular weight (Fig. 5g). Meanwhile, structural modelling of the TCF4 in complex with TEAD4 and DNA also suggest that these two transcription factors may co-bind to DNA with their DNA binding domains contacting each other without apparent steric hindrance, that is, TEAD4 and TCF4 may directly interact with each other on their co-bound DNA (Fig. 5h). From the modelled structure, the interaction between TEAD4 and TCF4 could strengthen or stabilize the DNA binding of TCF4 by helping to ‘wrap around' the DNA.

Given that the TEA domain of TEAD4 is required for its interaction with TCF4 but is dispensable for VGLL4 binding, we postulated that the TEA domain fragment would interfere with the inhibitory effect of VGLL4 on the Wnt/β-catenin signalling in a dominant-negative manner. We therefore co-transfected the TEA domain fragment together with VGLL4 in HCT116 cells. As shown in Fig. 5i,j, transfection of GFP-tagged TEA domain (termed as GFP-TEA) substantially abolished the inhibitory function of VGLL4 on Wnt/β-catenin signalling, according to TOP-flash transactivation and the mRNA levels of Wnt target genes Axin2 and CCND1; while the GFP control did not affect such activity of VGLL4. Altogether, these results indicate that the TEA domain of TEADs is required for VGLL4-mediated inhibition of Wnt/β-catenin signalling even though it is dispensable for binding VGLL4.

Collectively, these results indicate that TEAD4 and TCF4 can form a complex on Wnt target genes via a direct interaction between their DNA-binding domains, and that VGLL4 may bind to this complex to interfere the transactivation of TCF4.

### VGLL4 inhibits *in vitro* and *in vivo* colorectal cancer growth

**Figure 6:**
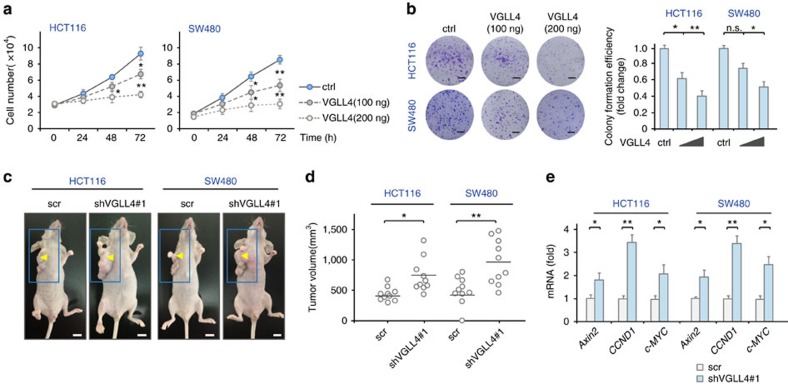
VGLL4 inhibits colorectal cancer cell growth. (**a**) VGLL4 inhibited proliferation of HCT116 cells and of SW480 cells. (**b**) VGLL4 inhibited colony formation of HCT116 and SW480 cells. Scale bar denotes 10 mm. (**c**,**d**) Knockdown of endogenous VGLL4 by shVGLL4#1 promoted xenograft tumour growth. Mice were photographed after being killed. Black bar denotes the mean value from one experiment. Scale bar denotes 10 mm. (**c**) Tumours harvested from each mouse were photographed before further processing. (**d**) The tumour volumes were measured for the mice from each group (ten mice). (**e**) mRNA levels of Wnt target genes in samples from panel d. Note: Data in **a**,**b**,**d** and **e** were from one experiment. Experiments we repeated two times. Unpaired *t* tests were used to compare the difference between groups. *P<0.05, **P<0.01, significant relative to control. n.s., no statistics significance.

It is well established that Wnt/β-catenin signalling is important for the proliferation of colon cancer cells. Therefore, by using ATPase and colony formation assays, we examined whether VGLL4 regulates the proliferation of HCT116 and SW480 cells. As shown by the ATPase assay, transient expression of VGLL4 suppressed in a dose-dependent manner the growth of HCT116 and SW480 cells, to ∼42% and 33%, respectively of the levels from an empty vector after 72 h of transfection (Fig. 6a). These results were subsequently confirmed by colony formation assay. We transfected VGLL4 in HCT116 and SW480 cells and counted the number of colonies after two weeks of G418 selection. The colony numbers of VGLL4-transfected HCT116 and SW480 cells were decreased to 36% and 52%, respectively, of the numbers obtained when the cells were transfected with an empty vector (Fig. 6b). Altogether, these observations indicate that VGLL4 suppresses proliferation of colon cancer cells.

We next evaluated the role of VGLL4 in tumour formation of HCT116 and SW480 cells by using a xenograft model of nude mice. As shown in Fig. 6c,d, stable lentiviral depletion of VGLL4 in HCT116 and SW480 cells significantly promoted tumour growth *in vivo* when compared with the control (scramble). Consistent with this observation, transcription of the Wnt target genes *Axin2*, *CCND1* and *c-MYC* was markedly enhanced in tumours in which VGLL4 was depleted (Fig. 6e). Taken together, these observations indicate that VGLL4 suppresses CRC tumour growth at least partially by inhibiting the Wnt/β-catenin pathway.

### A VGLL4-mimicking peptide inhibits CRC in *APC* ^*min/+*^ mice

**Figure 7:**
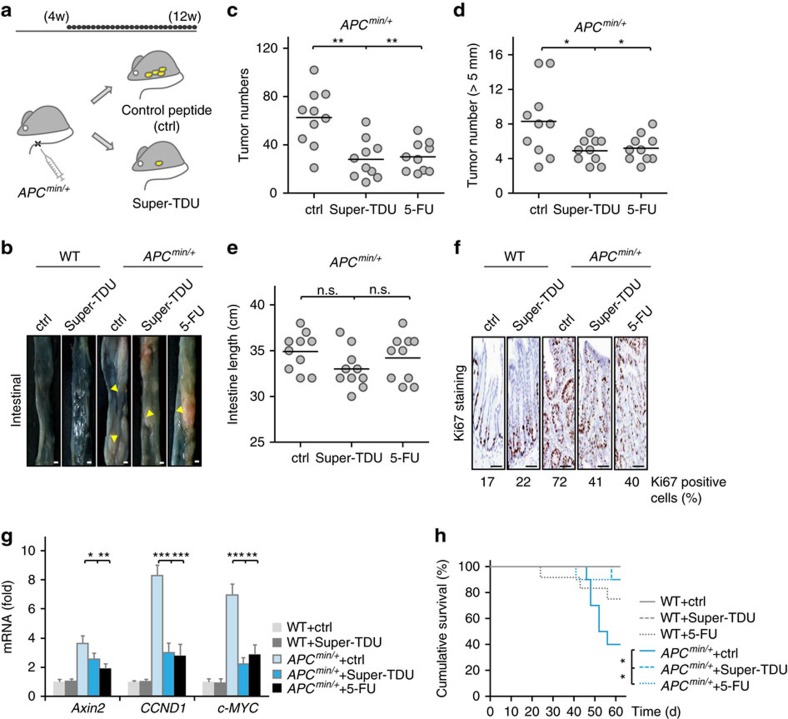
*APC*^*min/+*^ mice were sensitive to Super-TDU treatment. (**a**) Chart depicting the process used to treat *APC*^*min/+*^ mice with Super-TDU. *APC*^*min/+*^ and littermate wild-type (*APC*^*+/+*^) mice (aged 4 w) were randomized to receive 500 μg kg^−1^ (*n*=10) per day of Super-TDU peptide or control peptide (n =10) by tail vein injection. In addition, other mice were treated with 50 mg kg^−1^ 5-fluorouracil (5-FU) intravenously as a positive control (*n*=10). After eight weeks, the mice (aged 12 w) were killed, and their intestines (both the small intestine and colon) were extracted. (**b**) Pictures of the entire small intestine show large intestinal tumours. Arrows indicate adenomas. Scale bar denotes 1 mm. (**c**–**e**) Number of tumours (**c**), number of tumours (length>5 mm) (**d**) and intestinal length (**e**) were calculated. Data were from one experiment. Experiments we repeated two times. Unpaired *t* tests were used to compare the different between groups. (**f**) Ki67 staining of the intestines tissue of wildtype (WT) and *APC*^*min/+*^ mice treated with control, Super-TDU or 5-FU. The percentage of Ki67-positive cells were labelled below each group. Scale bar denotes 50 μm. (**g**) The relative expression of the Wnt target gene in intestinal tissue of wildtype (WT) and *APC*^*min/+*^ mice treated with control, Super-TDU or 5-FU. (**h**) Survival curves of the mice. Note that all mice survived for the first 25 days of treatment, and all WT mice treated with Super-TDU, as well as those treated with the control survived for the entire treatment period. Data were from one experiment. Experiments were repeated two times. Survival curves were calculated according to the Kaplan–Meier method; survival analysis was performed using the logrank test, *P<0,05, significant relative to control. Note: Data in **c**–**e** and **g** were from one experiment. Experiments were repeated two times. Unpaired *t* tests were used to compare the difference between groups. *P<0.05, **P<0.01, ***P<0.001, significant relative to control. n.s., no statistics significance (Supplementary Figs 7 and 8).

To further evaluate the anti-tumour effect of VGLL4 in CRC, we made use of a peptide that mimics the function of VGLL4 (termed as Super-TDU), which we developed previously for the study of Hippo-YAP signalling^31^. We first tested and confirmed that both HCT116 and SW480 cells were sensitive to treatment with the Super-TDU peptide (Supplementary Fig. 7). We then chose adenomatous polyposis coli (*APC*) mutant mice (*APC*^*min/+*^), a well-known mouse model for colorectal and intestinal cancer, to further corroborate the function of VGLL4 in CRC. *APC*^*min/+*^ mice at 4 weeks of age were treated with the Super-TDU peptide by tail vein injection for 8 weeks, and then the mice were killed (Fig. 7a). In parallel, as a positive control, mice were intravenously treated with 50 mg kg^−1^ 5-fluorouracil (5-FU), a conventional drug used in cancer therapy. Adenomas were counted manually, as described in the Methods section, using a dissecting microscope. As shown in Fig. 7b, *APC*^*min/+*^ mice exhibited significantly more and larger spontaneous adenomas than did wildtype control mice; while treatment with Super-TDU and 5-FU alleviated such situation. After treatment with the Super-TDU peptide, the number and average size of adenomas were significantly decreased, by ∼65%, as compared with those treated with a control peptide (Fig. 7c). Meanwhile, the number of tumours with a diameter larger than 5 mm was also reduced significantly in the Super-TDU-treated group (Fig. 7d). Since the intestinal length can be different for each individual mouse and be affected by health conditions including inflammation. Therefore, we also measured the length of the intestines. As shown in Fig. 7e, we did not find any significant difference among different groups of mice.

Subsequently, we performed Ki67 staining of the intestines. Consistent with the above observations, treatment with the Super-TDU peptide reduced the Ki67-positive cells by approximately 30% in *APC*^*min/+*^ mice, as compared with those treated with a control peptide (Fig. 7f). Next, we analysed the expression of Wnt target genes *Axin2*, *CCND1* and *c-MYC*. Treatment with the Super-TDU peptide efficiently inhibited the transcription of these genes in *APC*^*min/+*^ mice but had little effect in wildtype mice (Fig. 7g). Moreover, the Super-TDU peptide did not exhibit a detectable toxicity, that is, there were no deaths of wildtype mice treated with this peptide at the 500 μg kg^−1^ dosage that clearly displayed an anti-tumour effect, while there were two deaths of wildtype mice treated with 5-FU at a dosage that displayed a similar anti-tumour effect as did the Super-TDU peptide (Fig. 7h). Taken together, these observations support the proposal that VGLL4 functions as a CRC tumour suppressor at least partially through downregulation of Wnt/β-catenin signalling.

## Discussion

Tumorigenesis is frequently associated with dysregulation of a signalling network, rather than of a single pathway. Both Wnt/β-catenin and Hippo-YAP signalling pathways are critically involved in the initiation and development of multiple cancers including CRC. Recent studies indicate multi-point crosstalk between these two pathways, which is mainly concerned with the subcellular localization and protein stability of the key signalling effectors β-catenin and YAP. Our current study revealed VGLL4 as a tumour suppressor in CRC and highlighted its potential anti-cancer application as a targeted therapy aiming at both Wnt/β-catenin and Hippo-YAP signalling. Instead of altering the subcellular localization of YAP and β-catenin, VGLL4 coregulates Wnt/β-catenin and Hippo-YAP signalling by targeting the functional interplay between TEAD4 and TCF4 that is essential for target gene transcription (Supplementary Figs 8 and 9).

Previously, we and others identified VGLL4 as a natural antagonist of YAP that can directly compete with YAP for binding TEAD4 (refs 28, 29, 30, 31). In the current study, we showed that VGLL4 negatively regulates Wnt/β-catenin signalling in a TEAD4-dependent manner. Disruption of VGLL4–TEAD4 interaction abolished the inhibitory effect of VGLL4 on Wnt/β-catenin signalling. In particular, TEAD4 directly interacts with TCF4 through their DNA-binding domains; TEAD4 and TCF4 co-bind to target gene promoter to form a complex and facilitate TCF4 transactivation. As such, the TEAD4–TCF4 complex can promote the transcription of target genes that even does not contain classical TEAD4 consensus binding sites in their promoters. Meanwhile, the TEA domain of TEAD4 plays a critical role because it is required for both direct interactions with TCF4 and association with DNA. VGLL4 binds to the YBD domain of TEAD4 to form a complex with TCF4–TEAD4 and inhibit the transactivation of TCF4. Despite of many details to elaborate, one may propose a model in which Wnt and Hippo pathways are directly linked at the level of transcription factors TCF4 and TEAD4; binding partners of TEAD4 such as YAP and VGLL4 may influence TCF4 transactivation by targeting the TCF4–TEAD4 complex.

It is worth noting that most studies to date have pointed to YAP/TAZ in the regulation of the Wnt/β-catenin pathway, with the role of TEAD4 underappreciated in this process. It has been reported that the absence of Hippo signal significantly promotes the transcription of Wnt/β-catenin target genes including sox2 and snail2 (ref. 25). It appears that YAP-TEAD4 and β-catenin-TCF4 may cooperatively regulate at least a group of co-target genes. Our results now clearly indicate that TEAD4 can directly interact with TCF4 on Wnt target genes to facilitate transactivation of TCF4. Such cooperation between TEAD4 and TCF4 is likely disrupted by VGLL4 binding and thus conformational disturbance of the complex. This is despite that VGLL4 does not significantly alter the nuclear translocation of either YAP (refs 29, 30, 31) or β-catenin. In this regard, our study identified TEAD4 as an essential component for Wnt/β-catenin signalling, and uncovered a TCF4–TEAD4 complex as a key determinant for nuclear crosstalk between Wnt/β-catenin and Hippo-YAP pathways. VGLL4 can target the TCF4–TEAD4 complex to simultaneously suppress the transactivation of both TCF4 and TEAD4, representing a previously unrecognized type of mechanism for the coregulation of Wnt/β-catenin and Hippo-YAP signalling.

Interestingly, our study showed that the interaction between TEAD4 and TCF4 is direct, but also somehow sensitive to the presence of DNA, that is, they do bind each other directly, but DNA binding may further promote or stabilize their interaction. Actually, such DNA-facilitated transcription factor interaction/pairing could be a common theme of cooperation between transcription factors^27^. Besides being a molecular machinery of the crosstalk between Wnt and Hippo signalling pathways, it is also provoking to consider the TEAD4–TCF4 interplay as another example of transcription factor cooperation.

Colorectal carcinoma is the third most common type of cancer and the third most prevalent cause of death by cancer in the world^35^. Majority of CRCs harbour Wnt activating mutation. Currently it lacks targeted therapy aiming at the Wnt-activating mutations. Here we show that VGLL4 inhibits Wnt/β-catenin target gene transcription via its interaction with TEAD4, a downstream transcription factor of the Hippo-YAP pathway. VGLL4 was downregulated in CRC, with its expression level being inversely correlated with those of Wnt/β-catenin and Hippo-YAP target genes, indicating that VGLL4 acts as a tumour suppressor in CRC by co-regulating both signalling pathways. Moreover, a rationally designed VGLL4-mimicking peptide significantly suppresses CRC progression in mouse model (*APC*^*min/+*^ and xenograft) in which β-catenin is hyperactivated, exemplifying a potential targeted therapy pointing to the crosstalk node between Wnt and Hippo pathways. It remains to be determined whether VGLL4 functions as a turn-off checkpoint during the late stage of Wnt signalling, and whether there is another co-regulator of Wnt/β-catenin and Hippo-YAP signalling that also targets the TCF4–TEAD4 complex via a similar mechanism. Investigations in these regards should help advance the development of new strategies for treating cancer.

## Methods

### Collection of human colorectal cancer specimens

Thirty and five paired fresh colorectal carcinoma and normal tissue samples from patients, who underwent surgery between October 2010 and September 2012, were collected for quantitative RT-PCR and western blot analysis, respectively. On the day of tumour resection, the specimen was received in the laboratory immediately following surgery. The specimen was bathed in sterile saline in a sterile container. Research samples were collected during this dissection, including procurement of tissue for confirmation of diagnosis by pathology. The disease stage of each patient was classified or reclassified according to the 2009 AJCC staging system^36^. All samples collected and used were derived from patients who signed an informed consent approved by the institutional review board of local health department. All patients receiving treatment on this study were treated as part of a clinical protocol.

### Tissue microarray and immunohistochemical staining (IHC)

CRC and normal tissue microarray sections were prepared by Shanghai Outdo Biotech Co. Ltd. (Shanghai, China). This tissue array contains tissues from 40 human patients containing normal colons and 60 patients with CRC to examine the expression profiles of VGLL4 by IHC. For IHC, TMA sections were incubated with anti-VGLL4 antibody (1:50 dilution; ab140290; abcam). VGLL4 staining were scored by two independent pathologists, blinded to the clinical characteristics of the patients. The scoring system was based on the staining intensity and extent. Staining intensity was classified as 0 (negative), 1 (weak), 2 (moderate) and 3 (strong). Staining extent was dependent on the percentage of positive cells (examined in 200 cells) were divided into 0 (<5%), 1 (5–25%), 2 (26–50%), 3 (51–75%) and 4 (>75%). According to the staining intensity and the staining extent scores, the IHC result was classified as 0–1, negative (−); 2–4, weakly positive (+); 5–8, moderately positive (++) and 9–12, strongly positive (+++).

### Cell culture and antibodies

HEK293T cells, L cells (an immortalized mouse fibroblast cell line), SW480 cells and HCT116 cells were obtained from Shanghai Life Academy of Sciences cell library (Shanghai, China). All cells were grown in DMEM medium (Invitrogen, Carlsbad, CA), and maintained in culture supplemented with 10% heat-inactivated fetal calf serum, 100 ug ml^−1^ of penicillin, and 100 μg ml^−1^ of streptomycin at 37 °C with 5% CO_2_ in a humidified incubator (Thermo, Waltham, MA). During the study, all cell cultures were periodically tested for mycoplasma by using MycoAlert Mycoplasma Detection Kit (Lonza, Rockland, ME). For western blot, antibodies specific for VGLL4 (1:500, ab140290), TEAD4 (1:500, ab58310), Histone H3 (1:1,000, ab6002) and β-catenin (1:1,000, ab2365) were purchased from Abcam (Cambridge, UK); those for FLAG (1:5,000, M4439) and α-tubulin (1:2,000, T6199) were from Sigma (St. Louis, MO); and those for TCF4 (1:500, sc-166699), c-Jun (1:1,000, sc-4113) and GST (1:1,000, sc-138) were bought from Santa Cruz Biotechnology (Santa Cruz, CA).

### Plasmids

Mammalian expression vectors for VGLL4 and its HF4A mutant were described previously^31^. All lentiviral plasmids were constructed in a modified pLKO.1 vector. Two pairs of short hairpin RNA (shRNA) oligos of VGLL4 were designed and synthesized. For oligo-1 (from the coding sequence of VGLL4), forward oligo: 5′ CCGGGAGCCTGGGCAAGAAT TACAACTCGAGTTGTAATTCTTGCCCAGGCTCTTTTTG-3′; reverse oligo: 5′ AATTCAAAAAGAGCCTGGGCAAGAATTACAACTCGAGTTGTAATTCTTGCCCAGGCTC-3′. For oligo-2 (from the 5′-UTR sequence of VGLL4), forward oligo: 5′-CCGGAGGAGCTACTCAGCAACAATTGACTCGAGTCAATTGTTGCTGAGTAGCTCCTTTTTTG-3′; reverse oligo: 5′- AATTCAAAAAAGGAGCTACTCAGCAACAATTGACTCGAGTCAATTGTTGCTGAGTAGCTCCT -3′. A scramble DNA duplex was also designed as a control.

### siRNAs

Duplexes of siRNA targeting TEAD1, TEAD2, TEAD3, TEAD4, TCF4 and negative control were synthesized by Genepharma (Shanghai, China). The siRNA sequences are as following. For human TEAD1 (targeting its CDS sequence), the forward oligo used were 5′–GGCAUGCCAACCAUUCUUATT–3′, and the reverse oligo were 5′–UAAGAAUGGUUGGCAUGCCTT–3′. For human TEAD2 (targeting its CDS sequence), the forward oligo used were 5′–GCCAGAUGCAGUUGAUUCUTT–3′ and the reverse oligo were 5′–AGAAUCAACUGCAUCUGGCTT–3′. For human TEAD3 (targeting its CDS sequence), the forward oligo used were 5′–CAGCCACAUACAGGUUCUATT–3′, and the reverse oligo were 5′–UAGAACCUGUAUGUGGCUGTT–3′. For human TEAD4 (targeting its UTR sequence), the forward oligo used were 5′–GGAACAAACUGUGCCUGAATT–3′, and the reverse oligo were 5′–UUCAGGCACAGUUUGUUCCTT–3′. For human TCF4 (targeting its UTR sequence), the forward oligo used were 5′–GGGACAUGCAUGGAAUCAUTT–3′, and the reverse oligo were 5′–CCCUGUACGUACCUUAGUATT–3′.For negative control, the forward oligo used were 5′–UUCUCCGAACGUGUCACGUTT–3′, the reverse oligo were 5′–ACGUGACACGUUCGGAGAATT–3′.

### Transfection and luciferase assay

Transient transfection of cells was performed using Lipofectamine 2,000 from Invitrogen (San Diego, CA) according to the manufacturer's instructions. To select stable transfectants, the cells were transfected and incubated overnight, and then switched to a medium containing G418 (600 μg ml^−1^) for further incubation. The medium that contained G418 was changed every two to three days. After two weeks, isolated colonies began to appear. In three weeks, a pool of G418-resistant cells was obtained for further studies. Luciferase activities were determined using the Dual-Luciferase Assay System (Promega).

### Real-time PCR

Real-time PCR was performed on an Applied Biosystems Step Two Real-Time PCR System (Applied Biosystems) using the comparative Ct quantization method. Real-time PCR Master Mix (Toyobo) was used to detect and quantify the expression level of the target gene. GAPDH was used as an internal control. The primers used were as follows:

*hVGLL4*: 5′–AACTGCAACCTCTCGCACTG–3′ (F), 5′–GAGTGGGTGTCGCTGTTGAA–3′ (R);

*hAxin2*: 5′–CTGGCTTTGGTGAACTGTTG–3′ (F), 5′–AGTTGCTCACAGCCAAGACA–3′ (R);

*hAxin2* (ChIP): 5′–CGCCTTTGAAGTGCACAGT–3′(F), 5′–ACCCAGGTCCTGTTTCCAG–3′(R); *hCCND1*: 5′–GCTGCGAAGTGGAAACCATC–3′ (F), 5′–CCTCCTTCTGCACACATTTGAA–3′ (R); *hc-MYC*: 5′–GGCTCCTGGCAAAAGGTCA–3′ (F), 5′–CTGCGTAGTTGTGCTGATGT–3′ (R); *hGAPDH*: 5′–GACGTGAGCATGTACCCTAGC–3′ (F), 5′–GCGTAGCCATTCCAGTCCT–3′ (R); *mAxin2*: 5′- ATGAGTAGCGCCGTGTTAGTG -3′ (F), 5′- GGGCATAGGTTTGGTGGACT -3′ (R); *mCCND1*: 5′- GCGTACCCTGACACCAATCTC -3′(F), 5′- ACTTGAAGTAAGATACGGAGGGC–3′(R); *mc-myc*: 5′- ATGCCCCTCAACGTGAACTTC–3′ (F), 5′- GTCGCAGATGAAATAGGGCTG–3′ (R); *mGAPDH*: 5′–AATGGATTTGGACGCATTGGT–3′(F), 5′–TTTGCACTGGTACGTGTTGAT–3′(R). Note: F, forward; R, reverse.

### RNA microarray analysis

Microarray assay was performed by knockdown and overexpression of VGLL4 in HCT116 cells. In either case, the microarray data presented in the manuscript is average value from two replicates. Specifically, HCT116 were transfected with the indicated plasmids or siRNAs (2 biological repeats). Following 48 h transfection, RNA was extracted using TRIZOL Reagent (#15596-018#Life technologies, Carlsbad, CA) following the manufacturer's instructions and checked for a RIN number to inspect RNA integrity by an Agilent Bioanalyzer 2,100 (Agilent, Santa Clara, CA,). Qualified total RNA was further purified by RNeasy mini kit (#74106, QIAGEN, GmBH, Germany) and RNase-Free DNase Set (#79254, QIAGEN, GmBH, Germany). Total RNA was amplified and labelled by Low Input Quick Amp Labelling Kit, One-Color (#5190-2305, Agilent), following the manufacturer's instructions. Labelled cRNA were then purified by RNeasy mini kit (#74106, QIAGEN). Each slide was hybridized with Cy3-labelled cRNA and hybridized overnight to a Human Gene Expression 4 × 44 K microarray(#G2534, Agilent). After hybridization, slides were scanned by Agilent Microarray Scanner (#G2565CA, Agilent).

### Cell viability assay

Cells were transfected with the indicated plasmids. An ATP-based cell viability assay was used for detecting cell proliferation. The ATP content was measured in accordance with the protocol of the CellTiter-Glo luminescent cell viability assay kit (Promega). Briefly, 100 μl of assay reagent was added to the wells, and mixed for 2 min at room temperature. After 10 min, intracellular ATP content was measured using a multilabel luminescence counter (Envision, Perkin Elmer). Cell viability was calculated using the following equation: % Cell viability=(value (test)–value (blank)) × (value (control)−value (blank))^−1^ × 100.

### Soft agar colony formation

Cells were stable-transfected with indicated plasmids. A total of 10^4^ cells were seeded on soft agar in six-well plates, and colonies with a diameter of >0.05 mm were counted 14 days after seeding.

### Xenograft tumour formation

Six-week-old healthy male nude mice (BALB/cA-nu/nu) were obtained from the Shanghai Experimental Animal Center and maintained in pathogen-free conditions. During the tumour formation assay, cancer cell lines transfected with VGLL4 shRNA were injected into the flank of the mice (HCT116 1 × 10^6^; SW480 2 × 10^6^). Mice were killed after four weeks and tumour volumes were then measured. Double-blind study has been designed and followed during the animal assays. All animals were randomly assigned to treatment groups in all experiments and used in accordance with the guidelines of the Institutional Animal Care and Use Committee of the Institute of Biochemistry and Cell Biology. The approval ID for using the animals was No. 081 by the Animal Core Facility of SIBCB.

### Spontaneous adenomas model in *APC* ^*min/+*^ mice

*APC*^*min/+*^ mice on a C57BL/6J background were kindly provided by Professor Lin Li, and subjected to a 12-h light and 12-h dark cycle. Six-week mice (male:female=1:1) were injected once daily with 0.1 ml of Super-TDU in saline via the tail vein. Mice were randomized to receive either 50or 500 μg kg^−1^ per day of Super-TDU peptide or control peptide. In addition, mice were treated with 50 mg kg^−1^ 5-fluorouracil (5-FU) intravenously as a positive control.

### Confocal imaging

L cells were plated on coverslips in six-well plates and transfected with the indicated plasmids. Twenty-four hours later, cells were treated with or without Wnt3a for 2 h. Coverslips with the cells were washed once with PBS and fixed in 4% formaldehyde in PBS for 15 min. After permeabilization with Triton X-100 (0.25%) in PBS for 15 min, cells were blocked with PBS containing BSA (5%) for 1 h and then incubated with primary antibodies for 1 h. After three separate washes, cells were incubated with a secondary antibody for another hour and then stained with DAPI for 2 min. The coverslips were washed extensively and fixed on slides. Images were captured using a Leica laser scanning confocal microscope (Leica TCS SP2 AOBS).

### DNA preparation

The downstream sequence of SOX2 gene containing TEAD and TCF binding sites were amplified by PCR. Target DNA were detected by agarose gel and purified by Gel Extraction Kit (Tiangen). The primers used were as follows: SOX2 sense: TAATATAAATTGAATGAATAAGAG; SOX2 antisense: CTCATGCACACAAAGGCACTG.

### Protein expression and purification

Human TEAD4 (35–434, deletion 121–174), TEAD4 (TEA, 38–105), TEAD4 (YBD, 217–434), VGLL4 (203–256), TCF4 (8–411, deletion 66–325), TCF4 (8–54), TCF4 (326–411) and β-catenin were cloned into pET28a with a His-SUMO, MBP, GST and His tag respectively followed by a tobacco etch virus (TEV) protease cleavage site at the N terminus and expressed in *Escherichia coli* (*E. coli*) BL21 (DE3) CodonPlus cells. The protein expression was induced by 0.4 mM isopropyl β-D-thiogalactopyranoside at A_600_=0.8 in Terrific Broth medium and the cells were cultured for an additional 18 h at 16 °C. The cells were then harvested by using centrifugation and then resuspended in the lysis buffer (20 mM Hepes, 500 mM NaCl, 10% glycerol, 1 mM DTT, pH 7.5). The cell homogenates were lysed by using a high-pressure homogenizer (JNBIO3000plus) at 1,800 bar and cell debris was removed by centrifugation at 20,000*g* for 40 min. The soluble fraction was loaded onto a Ni or Glutathione Sepharose (GE Healthcare) or Amylose Resin (NEB) pre-equilibrated with lysis buffer and the proteins were eluted with an elution buffer (20 mM Hepes, 500 mM NaCl, 10% glycerol, 1 mM DTT, pH 7.5) plus 400 mM imidazole, 20 mM reduced glutathione or 20 mM maltose. Untagged proteins were generated by TEV protease treatment. Tagged proteins were concentrated and applied to a HiLoad 16/60 Superdex 200 pg column (GE Healthcare) pre-equilibrated with a buffer consisting of 20 mM Hepes, 100 mM NaCl, and 1 mM DTT, pH 7.5. Untagged TEAD4 was applied to the same column pre-equilibrated with a buffer consisting of 20 mM Hepes, 250 mM NaCl and 1 mM DTT, pH 7.5. The purity of the proteins was monitored by SDS–polyacrylamide gel electrophoresis (SDS–PAGE).

### Immunoprecipitation and immunoblot

For immunoprecipitation experiments, whole cell extracts were prepared with or without DNase I (10 μg ml^−1^) after transfection or stimulation, and incubated overnight with indicated antibodies (0.6 μg mg^−1^ protein) together with Protein A/G beads (Santa Cruz). Beads were then washed three times with lysis buffer, and immunoprecipitates were eluted with SDS loading buffer and resolved in SDS–PAGE gels. The proteins were transferred to PVDF membrane (Bio-Rad) and further incubated with the indicated antibodies.

### Pulldown assay

GST-TCF4 coupled on Glutathione Sepharose were mixed with different prey proteins or/and DNA at 4 °C for 1 h in 20 mM HEPES, 200 mM NaCl, and 1 mM DTT, pH 7.5, and then washed three times. The proteins bound on the resin were eluted by the same buffer but with 20 mM reduced glutathione also included. The input and output samples were loaded onto SDS-PAGE and detected by Coomassie blue staining.

### Chromation immunoprecipitation (ChIP)

HEK293T cells were suspended in 5 × volume of cell lysis buffer (10 mM Hepes-KOH, pH 7.8, 10 mM KCl, 0.1 mM EDTA, 0.1% NP-40) and incubated for 5 min on ice. The suspension was centrifuged at 700*g* for 3 min and then re-suspended in 3 × volume of cell lysis buffer using a 21-G syringe. The suspension was centrifuged at 700*g* for 3 min and the nuclei sedimented were resuspended in 9.5 ml PBS. The nuclei were initially fixed by adding 0.5 ml of 20 mM DSP and rotated for 30 min at 25 °C. The suspension was centrifuged at 190*g* for 3 min and nuclei were fixed with 1% formaldehyde for 10 min at 25 °C. The reaction was stopped by adding 0.5 ml of 2.5 M glycine and rotating it for 5 min. The suspension was centrifuged at 700*g* for 3 min and then resuspended in 0.3 ml nucleic lysis buffer (10 mM Tris–HCl, pH 7.5, 200 mM NaCl, 10 mM EDTA, 1% SDS) containing proteinase inhibitors. Lysates were sonicated to yield 300–1,000 bp DNA fragments. After elimination of cell debris by centrifugation, the sample was diluted with 1.8 ml of ChIP dilution buffer and was pre-cleared with 10 μl protein A-Sepharose (50% slurry) for 30 min at 4 °C with agitation. The sample (0.1 ml) was saved to assess input DNA. 2–5 μg of the antibodies TCF4 (sc-13027, Santa Cruz) and TEAD4 (ab58310, abcam) was incubated with the sheared cross-linked chromatin to immunoprecipitate the indicated complexes. Input and immunoprecipitated DNA were subjected to Sybergreen Q PCR cycles with primers overlapping the gene body and upstream and downstream regulatory regions of human *AXIN2*.

### Biolayer interferometry

Interaction analysis was performed using an Octet Red 96 instrument (ForteBio). Wild-type TCF4 was labelled by biotin in 20 mM Hepes pH 7.5, 100 mM NaCl, 1 mM DTT, and biotinylated proteins were immobilized on streptavidin (SA) biosensors and incubated with TEAD4 (TEA) proteins in 1 × kinetics buffer. Data were analysed using Octet Data Analysis Software 7.0 (ForteBio).

### Statistical analysis

Both cellular and animal studies tended to be underpowered. Estimated of sample size for a planned comparison of two independent means using a two-tailed test were undertaken using an online calculator and in the SAS statistical software package (9.1.3). Data are expressed as mean±s.d. or median and interquartile range for continuous variables and as frequencies and proportions for categorical variables. Continuous data were compared by using paired *t* test or unpaired *t* test. For correlation, the Spearman rank correlation was used for continuous variables. Fisher's exact test was used to identify statistical significance of correlation between VGLL4 staining (IHC) and clinicopathological factors. Survival curves were calculated according to the Kaplan-Meier method; survival analysis was performed using the logrank test. *P*<0.05 was considered to indicate a significant difference.

### Data availability

The RNA microarray data that support the findings of this study have been deposited in the NCBI Gene Expression Omnibus (GEO) with the accession code GSE81665. All other all relevant data are available from the authors.

## Additional information

**How to cite this article:** Jiao, S. *et al*. VGLL4 targets a TCF4–TEAD4 complex to coregulate Wnt and Hippo signalling in colorectal cancer. *Nat. Commun.* **8,** 14058 doi: 10.1038/ncomms14058 (2017).

**Publisher's note**: Springer Nature remains neutral with regard to jurisdictional claims in published maps and institutional affiliations.

## Supplementary Material

Supplementary Information Supplementary Figures

## Figures and Tables

**Table 1:** Clinical significance of VGLL4 expression in CRC.

**Groups**	**VGLL4 expression**				***n***	**Positive (%)**	***P*value (*Fisher's test*)**
	**−**	**+**	**++**	**+++**			
*Sex*							
Male	9	10	12	5	36	75.0	1.000
Female	6	6	8	4	24	62.5	
							
*Age*							
<60	8	7	8	3	26	69.2	0.7745
>=60	7	9	12	6	34	79.4	
							
*Tumour Size*							
pT1+pT2	4	7	14	7	32	87.5	0.0278*
pT3+pT4	11	9	6	2	28	60.7	
							
*Lymph node metastasis*							
N0+N1	5	12	16	7	40	87.5	0.0230*
N2+N3	10	4	4	2	20	50.0	
							
*Distant metastasis*							
M0	7	10	17	8	42	83.3	0.0514
M1	8	6	3	1	18	55.5	
							
*Tumour stage*							
I+II	4	8	15	6	33	87.9	0.0321*
III+IV	11	8	5	3	27	59.3	
Total	15	16	20	9	60		

^*^Statistically significant by using *Fisher's exact test*, *P*<0.05.
