# Supplementary material for: VGLL4 targets a TCF4–TEAD4 complex to coregulate Wnt and Hippo signalling in colorectal cancer
Source: Nat Commun. 2017 Jan 4;8:14058. doi: 10.1038/ncomms14058 (PMC5216127; doi:10.1038/ncomms14058)
Supplement: Supplementary Information — Supplementary Figures [file ncomms14058-s1.pdf]

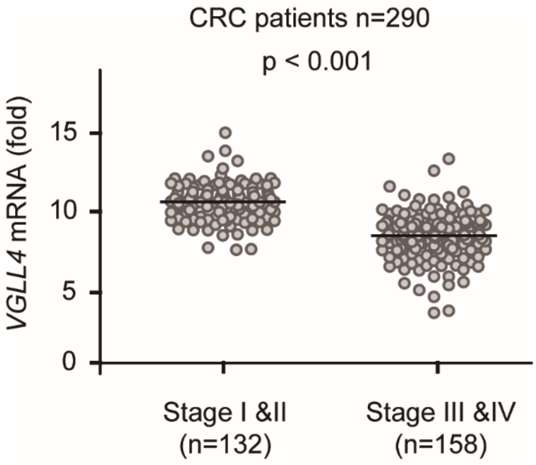

Supplementary Figure 1. Downregulation of VGLL4 in CRC. Relative VGLL4 mRNA levels in CRC patients of different tumor stages from GSE14333 dataset. Black bar denotes mean value. *Unpaired t tests* were used to compare the different between groups.

Related to **Figure 1.**

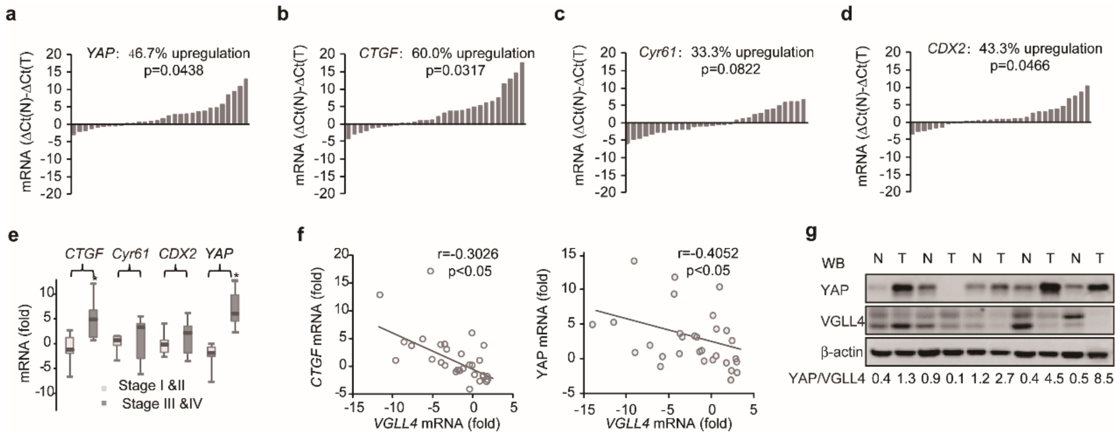

Supplementary Figure 2. Upregulation of Hippo signaling in CRC. (**a-d**) mRNA levels of *YAP* and YAP target genes in CRC tissue. Relative mRNA levels of *YAP* (**a**), *CTGF* (**b**), *Cyr61* (**c**) and *CDX2* (**d**) were analyzed. Experiments we repeated two times. Paired *t* tests were used to compare the different between paired tissue samples. (**e**) Box plots of *YAP* and YAP target genes levels at different tumor stages. Data are expressed as median and interquartile range (IQR). Experiments were repeated two times. *p < 0.05, significant relative to control. (**f**) Scatter plot between *VGLL4* and *CTGF* or *YAP* mRNA levels in CRC samples by applying Spearman's correlation. Experiments were repeated two times. (**g**) Levels of VGLL4 protein in five human colorectal carcinomas and their paired adjacent normal tissues were analyzed. Related to **Figure 2.**

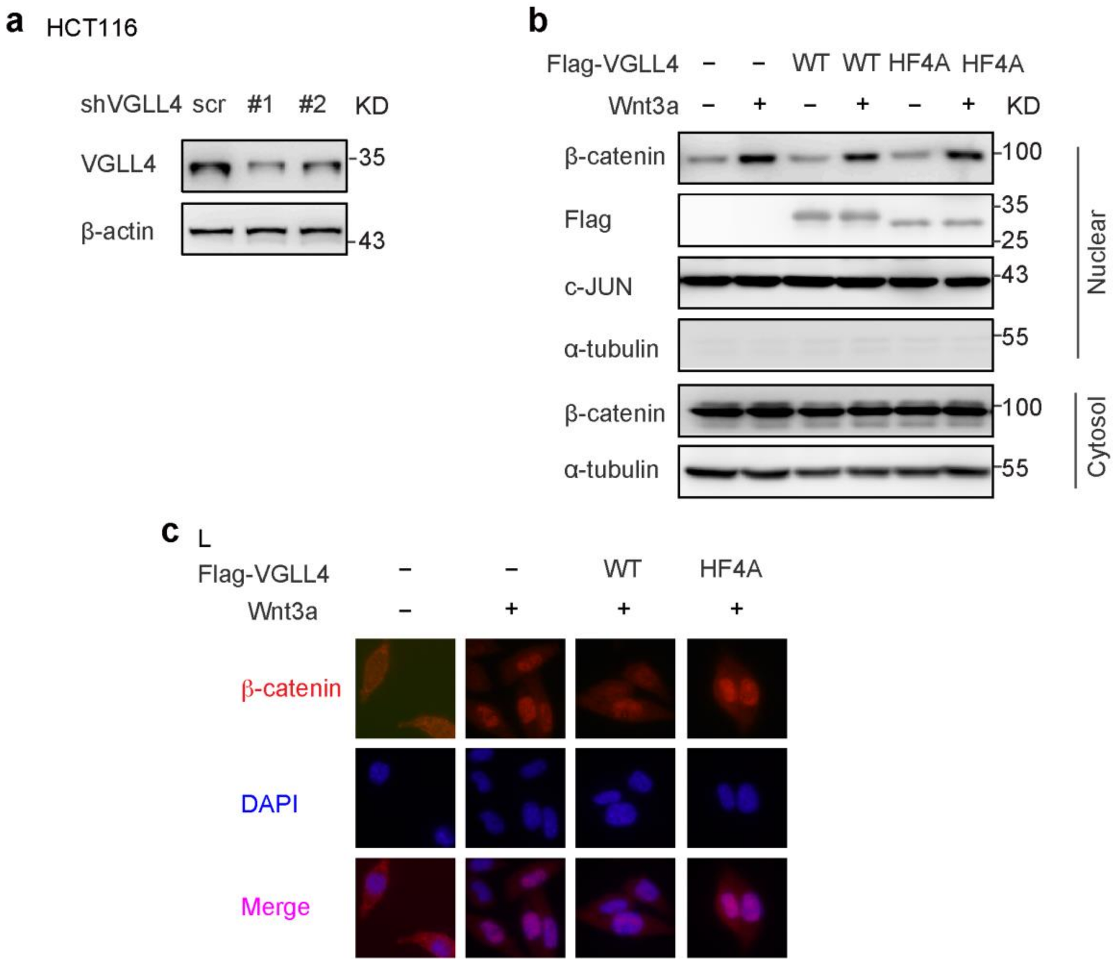

Supplementary Figure 3. Effect of VGLL4 on nuclear translocation of β-catenin. (**a**) Knockdown efficiency of two parallel VGLL4shRNA. (**b,c**) Nuclear translocation of β-catenin in cells transfected with VGLL4 or it's mutant. (**b**) Immunoblotting of nuclear β-catenin in HEK293T cells. (**c**) Representation images of β-catenin staining in L1 cells. (**b,c**) Relative activity of TEAD4 luciferase reporter (**b**), and mRNA levels of YAP target genes.

Related to **Figure 3.**

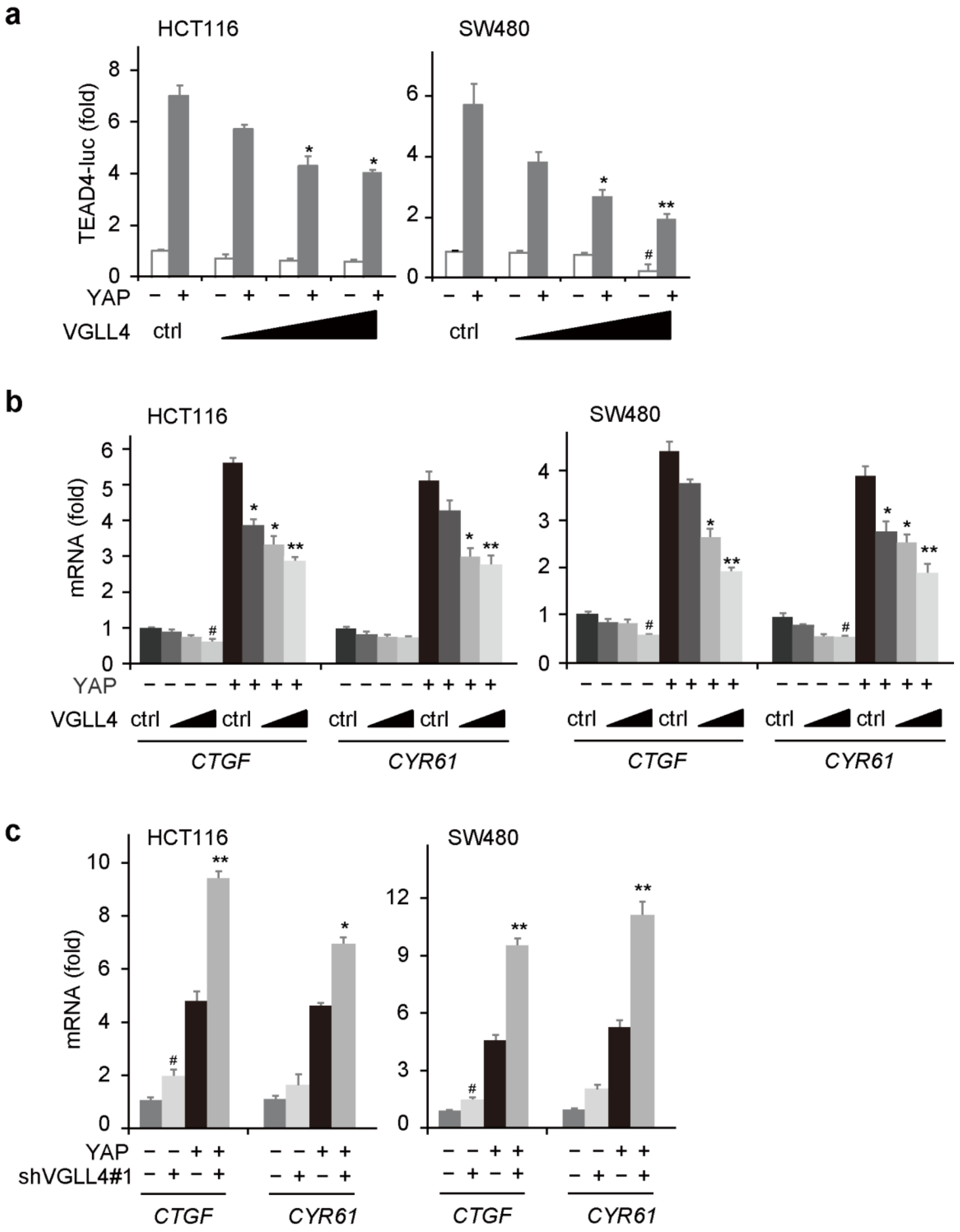

Supplementary Figure 4. Effect of VGLL4 on YAP signaling. (**a**,**b**) Relative activity of TEAD4 luciferase reporter (**a**), and mRNA levels of YAP target genes (**b**) in HCT116 and SW480 cells after transfection with VGLL4 in the absence or presence of YAP. (**c**) mRNA levels of YAP target genes in HCT116 and SW480 cells after transfection with shVGLL4 in the absence or presence of YAP. *Note:* Data represent the mean±S.D. from one experiment. Experiments we repeated three times. Unpaired *t* tests were used to compare the different between groups. $*p < 0.05$, $** p < 0.01$, significant relative to YAP-transfected group. $^{\#}p < 0.05$, significant relative to empty

vector-transfected group.
Related to **Figure 3.**

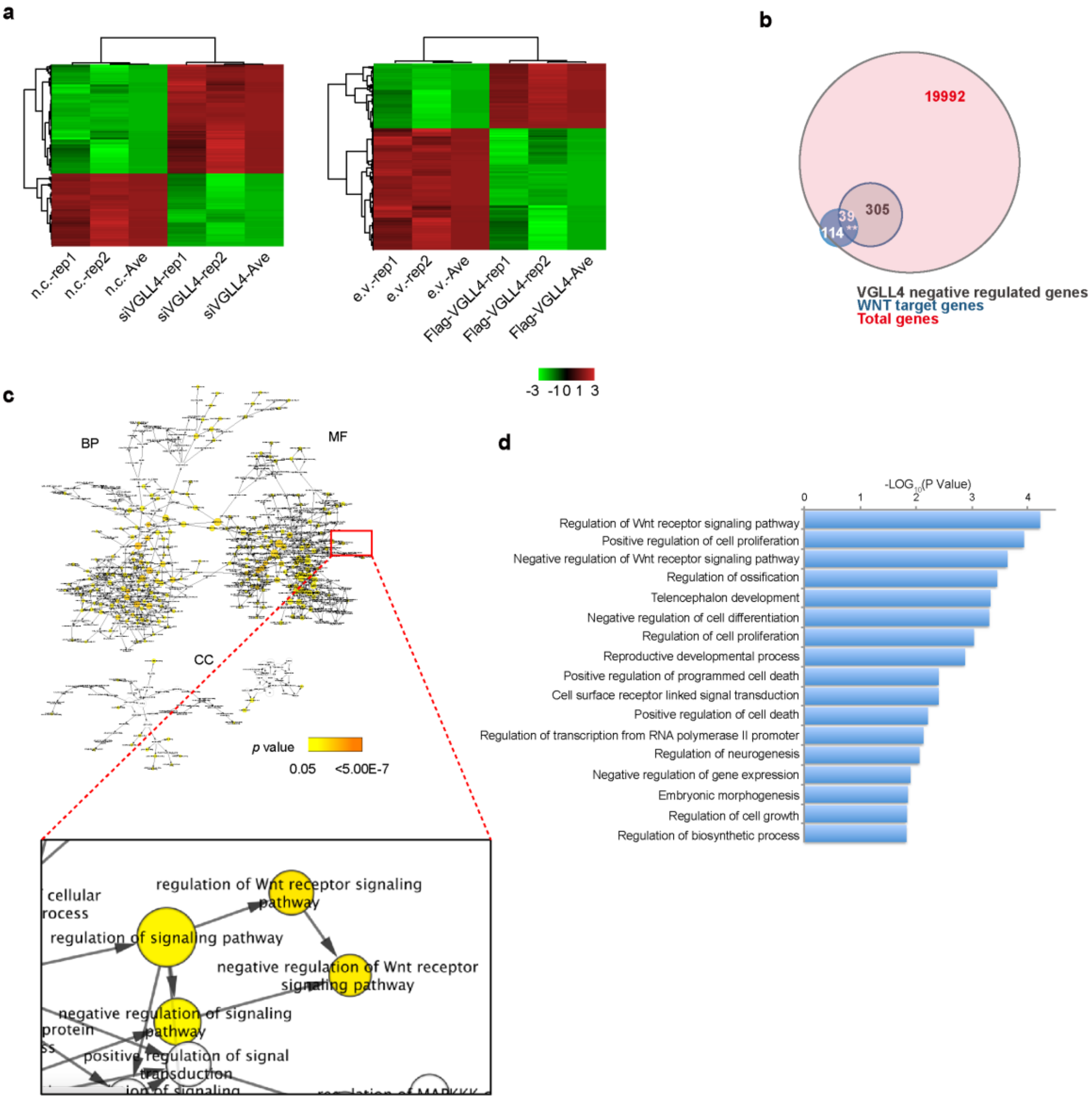

Supplementary Figure 5. Microarray and GO analyses in HCT116 cells. (**a**) Heat map of array based genome wide mRNA expression analysis. Hierarchical clustering analysis of significant changed genes that are very different between *n.c.* and *siSUN* groups was carried out. (**b**) Schematic illustration of the microarray data. *Note:* asterisk indicates that the ratio of VGLL4 negatively regulated Wnt target genes (39 out of 114, 34.2%) is statistically higher than the ratio of VGLL4 negatively regulated total genes expressed in HCT116 cells (305 out of 19992, 1.5%). (**c,d**) Hierarchy of biological process (BP), molecular function (MF), or cell colocalization (CC) or pathways defined in the GO or KEGG database.
Related to **Figure 3.**

a

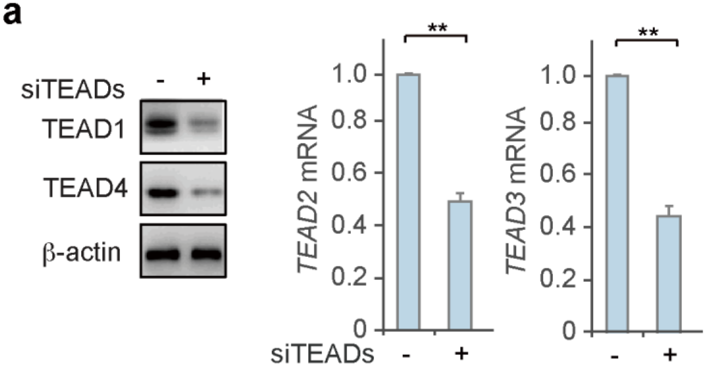

b

Gene Symbol	siTEADs	Gene Symbol	siTCF4
Axin2	**	*Axin2*	***
CCN1	*	*CCN1*	n.s.
CCND1	***	*CCND1*	**
DKK1	*	*DKK1*	**
DKK3	*	*DKK3*	*
EDN1	*	*EDN1*	*
FGF20	*	*FGF20*	***
LEF1	***	*LEF1*	***
MSX1	*	*MSX1*	-
MYC	**	*MYC*	***
ADM2	**	*ADM2*	**
ENC1	n.s.	*ENC1*	-
TNFRSF19	**	*TNFRSF19*	-
TNFRSF21	n.s.	*TNFRSF21*	*

Supplementary Figure 6. Functional interplay between TEADs and TCF4. (**a**) Knockdown efficiency of TEADs (1-4). **p < 0.01, significant relative to negative control group. (**b**) ChIP assay showing TCF4/TEAD4 binding to the indicated genes' promoters in 293T cells transfected with control siRNA, TEADs siRNAs or TCF4 siRNA. Left: siTEADs, ChIPed with TCF4 antibody. Right: siTCF4s, ChIPed with TEAD4 antibody. Experiments we repeated two times. Unpaired *t* tests were used to compare the different between groups. *p < 0.05, ** p < 0.01, *** p < 0.001, significant relative to control siRNA group. n.s., no statistical significance.

Related to **Figure 4.**

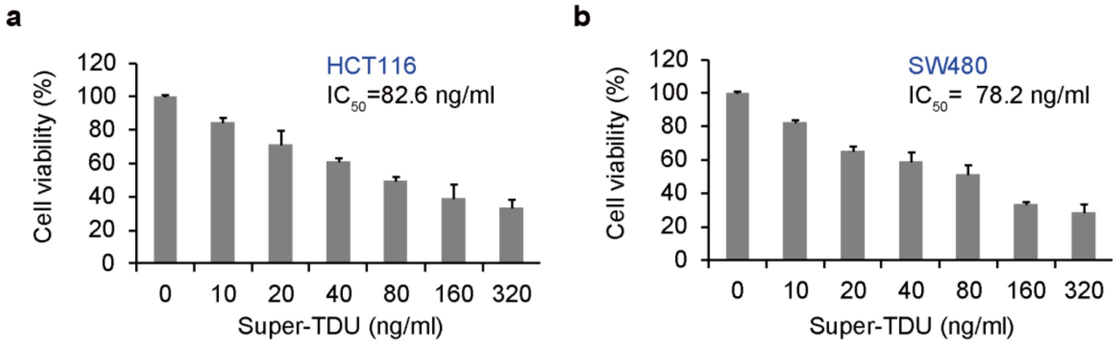

Supplementary Figure 7. Super-TDU Treatment of HCT116 and SW480 Cells. (**a**) Cell viability of HCT116 cells after treatment with Super-TDU. (**b**) Cell viability of SW480 cells after treatment with Super-TDU. Data represent the mean±S.D. from one experiment. Experiments we repeated three times. To calculate IC_{50}, linear regression was used.

Related to **Figure 7.**

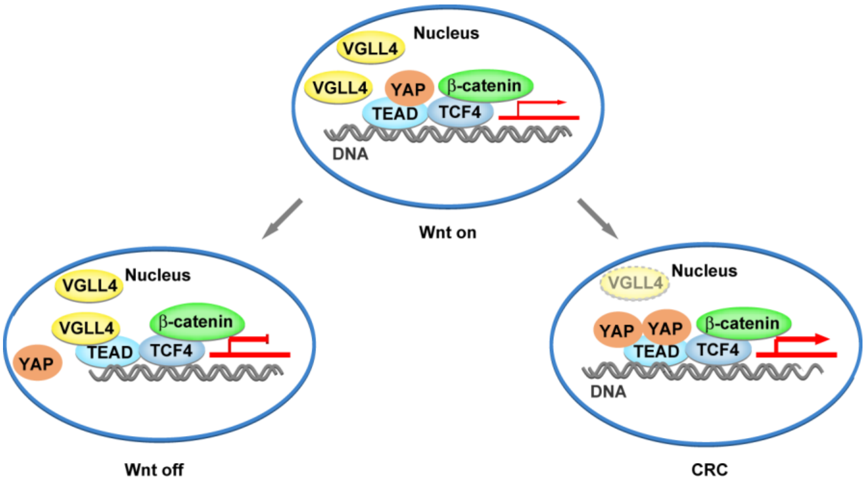

Supplementary Figure 8. Model for VGLL4 Regulation of TCF4 and TEAD4. TEAD4 directly associates with TCF4 to facilitate its transactivation. VGLL4 regulates Wnt/β-catenin signaling by targeting the TEAD-TCF4 complex to interfere the transactivation of TCF4 during Wnt3a-induced β-catenin signaling. VGLL4 is downregulated in CRC in which Wnt/β-catenin signaling is hyperactivated.

Related to **Figure 7**.

Figure 5c

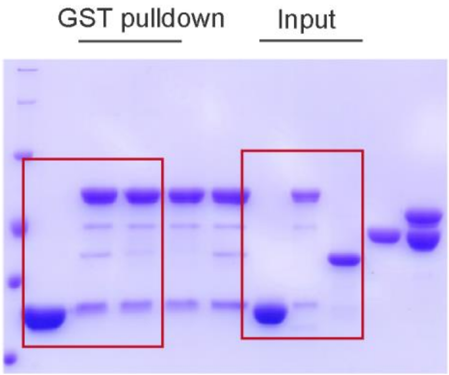

Figure 5g

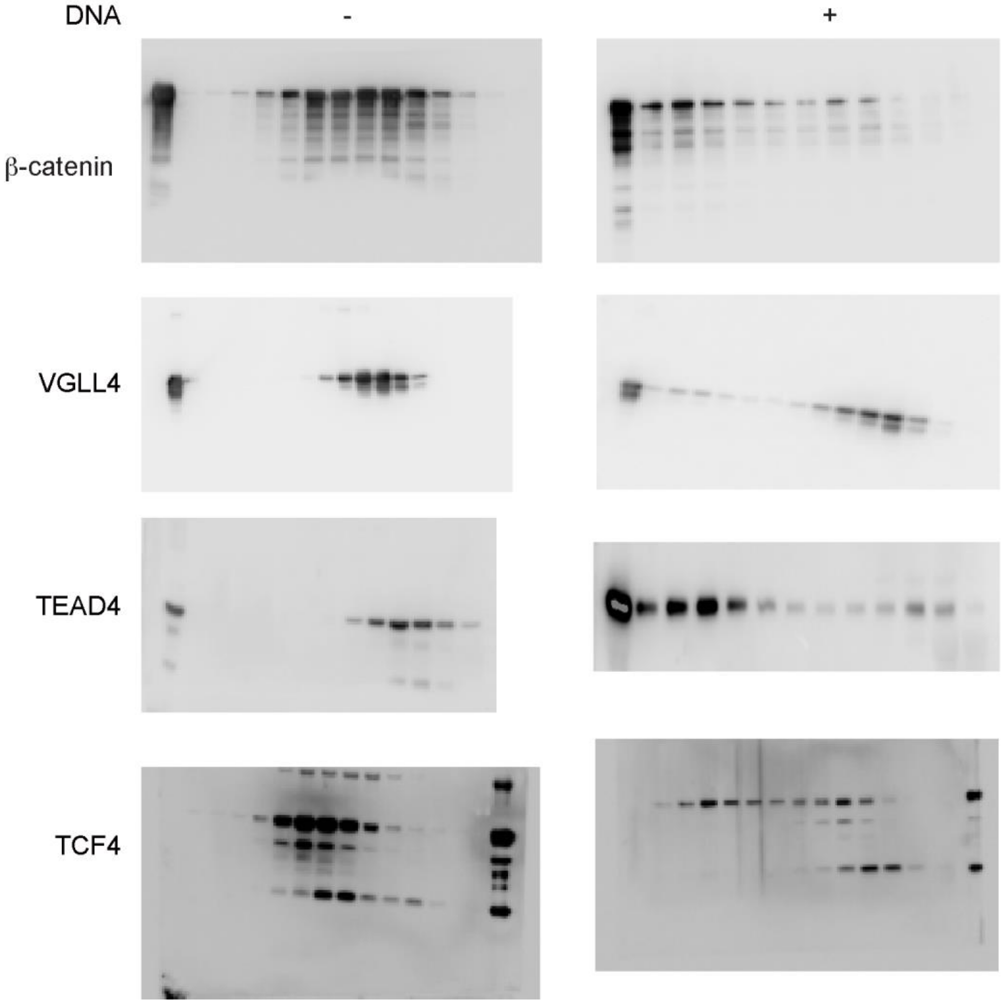

Supplementary Figure 9. Raw images used in Figure 5c and 5g.
